# An Analysis of Large-Scale Forced Migration in Africa

**DOI:** 10.3390/ijerph16214210

**Published:** 2019-10-30

**Authors:** Murat Bayar, Mustafa M. Aral

**Affiliations:** 1 Institute for Eastern and African Studies, Social Sciences University of Ankara, Ankara 06030, Turkey; 2 Department of Civil Engineering, Bartin University, Bartin 74100, Turkey; mmaral@live.com

**Keywords:** climate change, violence, human security, public health, migration, Africa

## Abstract

In this paper, human security-related causes of large-scale forced migration (LSFM) in Africa are investigated for the period 2011–2017. As distinct from the conventional understanding of (national) security, human security involves economic, public health, environmental and other aspects of people’s wellbeing. Testing various hypotheses, we have found that civil and interstate conflicts, lack of democracy and poverty are the most important drivers of mass population displacements, whereas climate change has an indirect effect on the dependent variable. As a policy tool, foreign aid is also tested to see if it lowers the probability of LSFM. Our findings have implications for policy planning, since the conventional understanding of security falls short of addressing LSFM without taking various aspects of human security into account.

## 1. Introduction

The aim of this study is to investigate factors behind forced migration in Africa. Our underlying assumption is that large-scale forced migration (LSFM) is a special case of population displacement, since it affects a sizable portion of a country’s population (at least 5 percent in our study). Africa constitutes our spatial parameter, because the continent had (as of February 2019) 6.3 million refugees and 14.5 million internally displaced persons (IDPs), who make up more than a third of all forcibly displaced people in the world [1]. The literature indicates that the likely causes of mass migration include socioeconomic, environmental, political and health-related factors, most of which can be studied under the concept of human security [2].

Since LSFM involves a sizable portion of a country’s population, it is more likely to transcend national borders and put pressure on transit and destination countries than small-scale migration. For instance, the European Union (EU) countries received a total of 1.3 million refugee claims in 2015 alone. In response, the EU Emergency Trust Fund for Africa has been created with financial and non-financial resources amounting to 3.39 billion euros with the purpose of assisting origin countries [3]. This funding initiative is part of the European Agenda on Migration, which focuses on “addressing the refugee crisis” and “managing external borders” [4] (p. 1).

African countries provide rich insights associated with various aspects of human security and LSFM. For instance, Cameroon is vulnerable against destabilizing cross-border effects from Nigeria and the Central African Republic, both of which are struggling with internal violence (Boko Haram terrorism and civil war, respectively). Furthermore, Cameroon is subject to the detrimental effects of sea level rise and also a major country of origin for the “Western migration route” (ending up in Spain). Ethiopia is another critical case for migrants originating in Eritrea and Somalia, which are major countries of origin for the “Central migration route” (ending up in Italy) [5].

Conducting a quantitative analysis of 48 African countries for the period 2011–2017, we investigate the impact of human security on forced migration in this study. We test climatic conditions, civil and interstate violence, political institutions, life expectancy (as a proxy for public health) and poverty as potential factors affecting population displacements. Furthermore, the role of foreign aid in alleviating these pressures is tested in a separate model, since this policy tool has been employed by several external actors, including the EU. Overall, our findings indicate that violent conflicts, authoritarian regimes and poverty are the primary causes of LSFM in Africa. Climate risk has an indirect effect on the dependent variable, and foreign aid falls short of alleviating the migration challenge.

## 2. Literature Review: Human Security and Forced Migration

Developed by the United Nations Development Programme (UNDP), the concept of human security stresses economic, environmental, health, food, personal/community and political aspects of people’s wellbeing [6]. Accordingly, the human development index introduced by UNDP has expanded the study of development from mere economic terms to 13 different dimensions [7].

Supported by the UN, the Commission on Human Security (CHC) has defined human security as *“… creating political, social, environmental, economic, military and cultural systems that together give people the building blocks of survival, livelihood and dignity*” [2] (p. 4). Human security and national security are not mutually exclusive, since a failed state would largely be incapable of providing essential provisions for its citizens. Yet, these two concepts are not fully compatible, because undemocratic states can protect their national borders while ripping their citizens of their personal and community resources. To note, human security has also been questioned for the tangibility of its contributions to the development debate, especially from an environmental perspective [8].

The study of forced migration requires that we clarify the differences between various migrant categories. The International Organization for Migration (IOM) has defined “migrant” as, “*any person who is moving or has moved across an international border or within a State away from his/her habitual place of residence, regardless of (1) the person’s legal status; (2) whether the movement is voluntary or involuntary; (3) what the causes for the movement are; or (4) what the length of the stay is*” [9].

The IOM has defined forced migration as, “*A migratory movement in which an element of coercion exists, including threats to life and livelihood, whether arising from natural or man-made causes (e.g., movements of refugees and internally displaced persons as well as people displaced by natural or environmental disasters, chemical or nuclear disasters, famine, or development projects)*” [10]. In parallel, “refugee” is someone “who has been forced to flee his or her country because of persecution, war or violence”, and “asylum seeker” is someone “whose request for sanctuary has yet to be processed” [11,12]. Although the IOM’s definition of “migrant” comprises “refugee” and “asylum seeker”, the legal framework of different countries can actually make a vital distinction for these categories. While refugees can be provided with some socioeconomic rights and a path to full citizenship, asylum seekers are not granted these options.

Noting the legal and conceptual differences between various migrant categories, we pool all refugees, asylum-seekers and other types of internally and externally displaced persons (but not voluntary migrants, as measured by the UN High Commissioner for Refugees—UNHCR) together for each country-year in this study. As discussed in the methodology section, our underlying assumption is that total number of forced migrants amounting to at least 5 percent of a country’s population constitutes a special case for origin countries, although these people may be classified differently in destination countries. For instance, Turkey considers forced migrants coming from Europe as refugees, whereas it considers those coming from “East” (including Africa) as asylum seekers [13] (p. 21). We assume that these legal differences have no theoretical implications for the purposes of this study, because we are investigating the relationship between our variables for each origin country, not for destination or transit countries.

The literature highlights international wage differentials, policies of origin and destination states, poverty and violent conflict among the drivers of human migration [14,15,16]. Simply put, repressive (e.g., against ethnic/religious minorities) home governments push, and welcoming (e.g., through multicultural policies) destination countries attract migrants. Not surprisingly, migrants flow from poor and/or conflict-ridden countries to relatively developed and stable states. Moreover, adverse environmental factors, such as drought, flood and inundation, increase the likelihood of conflict and migration. For example, Gleick has attributed the mass uprising and the subsequent civil war in Syria during the so-called Arab Spring to a prior collapse in food output due to several years of drought [17].

Given that several African economies rely heavily on agricultural production, their exposure to extreme weather conditions (whether causing drought, heavy rainfall or other extreme events) makes an adverse effect on the livelihoods of millions of people. Sea level rise poses another threat on both populations and commercial activities of coastal cities. Taken together with “poverty traps” that have strangled the continent for decades [18], these challenges are among the potential causes of large-scale human migration in Africa. Below we review various aspects of human security, as well as foreign aid as a potential remedy for these challenges.

### 2.1. Climate Change

The Intergovernmental Panel on Climate Change (IPCC) has pointed out that the most relevant phenomena affecting climate variability in Africa are monsoons, ENSO, Indian and Atlantic Ocean sea surface temperatures (SSTs), the atmospheric Walker circulation and the Mediterranean climate [19]. Climate change accelerates land degradation, particularly in low-lying coastal areas, river deltas and drylands. For example, during the period 1961–2013, the annual area of drought has increased, on average by slightly more than 1 percent per year with large inter-annual variability, and about 500 million people were living within areas that experienced desertification in 2015 (mostly South and East Asia, the Sahel region and the North Africa) [20]. The variations in Indian Ocean SST are also recognized as the dominant driver of East African rainfall and climate variability. Variability in Southern Africa’s climate is strongly influenced by its adjacent oceans, whereas variability in North Africa is characterized by the Mediterranean climate [20].

The projected increases in population and income, combined with changes in consumption patterns, will lead to increased demand for food, animal feed and water in Africa by 2050 in all shared socio-economic pathways (SSPs). Furthermore, the population of the African continent is estimated to be around 4.2 billion people by year 2100. These changes will have implications for land-use change, food insecurity, water scarcity, terrestrial GHG emissions, carbon sequestration potential and biodiversity. SSPs with greater cropland expansion will result in larger declines in biodiversity [19]. Compared to other regions, West Africa has a high number of people vulnerable to increased desertification, yield decline and water scarcity [19,20].

Thus, anthropogenic climate change is already threatening human security in Africa: Sahel countries (e.g., Sudan) may lose as much as half of their agricultural output due to worsening drought. East Africa may face an expansion of malaria-stricken areas because of increased rainfall and humidity, and West African countries (e.g., Ghana and Gambia) are threatened by sea level rise [19,21]. Ironically, Africa is warming faster than the rest of the world while contributing to greenhouse gases less than Eurasia and the Americas. Furthermore, the continent is negatively affected by the mitigation policies of developed countries in regard to commodity exports [22]. Overall, Busby et al. have looked into climate-related hazards, political violence, population density, household/community resilience and governance and mapped highly vulnerable sub-national areas as large swathes of West Africa, Central Africa and East Africa [23].

Several African countries are economically underdeveloped and lowly ranked at the human development index [7]. Consequently, they have relatively low adaptive capacity against climate change, which is likely to intensify LSFM. The World Bank has projected that there will be about 28 million “climate migrants” from the continent by 2050 even in the “more climate- friendly” scenario, and this number goes up as much as 71 million in pessimistic (high emission, low mitigation and adaptation) scenarios [24]. Since these estimates indicate a much higher number of forced migrants than the existing figures, the outcome would be devastating for all stakeholders, except for human traffickers.

In projections of the 21st century and beyond, the atmospheric models produce both significant drying and moistening of the continent yielding necessary policy adjustments [21]. In summary, considering the physical understanding of the processes involved and the expected large-scale climate evolution, the IPCC has concluded that the entire continent of Africa will most probably continue to warm during the 21st century with diverse effects on human populations [18,19,20,21]. The challenge taken in this study is to provide a quantitative analysis of the effects of this diverse variability on LSFM in the continent.

### 2.2. Economic Development and Foreign Aid

When African countries declared independence from colonial empires in the post-World War II era, they found themselves with little or no infrastructure available for manufacturing, education or healthcare, nor did they have much experience with running a modern state apparatus. Even Ghana, which inherited relatively good schools from the British Empire in 1957 and produced two-thirds of the world’s cocoa, faced several military coups and was still a poor country in the 1990s [25]. The World Bank has indicated that 24 of the 30 countries in the world with annual per capita incomes (GDP per capita, current international USD) less than 3000 USD are in Africa, and these figures do not even include the conflict-ridden countries like Somalia and South Sudan [26].

Since income per capita is a limited indicator of development, the multidimensional poverty index (MPI) was introduced in 2015 to measure “people’s deprivations” across education, health, and living standards (p. v). Employing the MPI, the Islamic Development Bank (IDB) and the University of Oxford have found that 62 percent of the people living in 22 IDB member countries in Sub-Saharan Africa are multidimensionally poor, and Niger fares worst with 89 percent of its citizens [27]. Furthermore, “heavily indebted poor countries” are mostly located in Africa and they have the highest (average) population growth rate (2.8 percent) among all economically defined clusters in the world [28]. In other words, several African countries are constrained by more than one poverty trap [18].

Considering the developmental challenges faced by young African countries, the United States (US) and the Soviet Union introduced foreign aid as a panacea starting as early as the 1950s. The US approach was based on its (then) recent successes with the Marshall Plan in Western Europe. In fact, France, West Germany and several other recipient states managed to rebuild their infrastructures and revived their industrial machines in just a couple of years despite their heavy losses in World War II [29]. Thus, the simple logic followed, aid as an international policy tool could also be used in the “Third World” in order to gain as many allies as possible.

Here, it is important to distinguish military aid from official development assistance (ODA), which is concessional (at least 25 percent grants) in character and involves the goal of promoting economic development and welfare of developing states [30]. For the purposes of this study, we use the terms foreign aid and ODA interchangeably while excluding military aid. Foreign aid has typically been justified by the strategic and economic interests of donor countries (e.g., US food assistance yielded higher agricultural employment for American farmers), and the historical responsibilities and moral obligations of the wealthy towards the poor [31].

Although most aid has typically flowed from “North” (i.e., developed countries) to “South” (i.e., developing countries), south-south cooperation has accelerated since the early 2000s. In his pioneering study on south-south cooperation, Bobiash investigated 14 south-south and two north-south aid cases in Senegal, Guinea-Bissau and Ghana. A career diplomat with strong academic credentials, he rightfully put, “*there is a strong subjective factor in aid evaluation, regardless of how ‘objective’ evaluation criteria and methodology appear to be*” (p. 25). Bobiash concluded that technology transfer was particularly successful between south-south partners due to their shared developmental and climatic conditions [32]. China and Turkey are among prominent southern donors with relatively less hierarchical relations with African recipients [33].

While foreign aid has proved successful in some cases [34], it has generally produced mixed, if not adverse results in promoting economic development [35,36]. Azarnert has demonstrated that the more a country is dependent on foreign aid (as a percentage of Gross National Income), the higher its fertility rates and the lower its educational attainment, thus, the more it gets stuck in poverty. His mathematical model has shown that, aid per child (as opposed to aid per adult) actually promotes a higher number of children, despite its negative impact on the average level of education [37].

Overall, the conflicts of interest between donors and recipients are commonly stated as a major reason behind the failure of foreign aid packages. Accordingly, the World Bank has called development agencies to become more selective, knowledge-based, self-critical, better coordinated and focus on systemic change as a criterion for success [38].

### 2.3. Public Health

The World Health Organization (WHO) has projected that an additional 250,000 human deaths per year may occur between 2030 and 2050 due to climate-related malnutrition, malaria and other health problems. It is expected that these problems will hit developing countries with poor health infrastructures the worst [39]. According to the WHO COP24 (Conference of the Parties) special report, climate-resilient health systems must be multifaceted with sustainable infrastructure (e.g., essential medical products and technologies), risk monitoring, emergency preparedness and health financing. However, the report has pointed out that only a small fraction (less than 1 percent) of multilateral climate finance has been directed into health projects [40].

Largely linked to climate, the public health challenges in Africa include (but is not limited to) undernutrition, meningitis, malaria, dengue fever and cholera. The WHO has pointed out, “Optimal physical growth and cognitive development are founded on maternal and child nutrition in the first 1000 days, with long-term health and economic implications for individuals and nations”. Nevertheless, undernutrition continues to be a widespread problem in Africa with 47 countries having more than 30 percent rates of childhood stunting (measured by a low score on height-for-age) [41]. Furthermore, Lloyd, Kovats and Chalabi have estimated that undernutrition caused by climate change will increase severe stunting among children in Sub-Saharan Africa by 23 percent in 2050 [42].

Brooks, Adger and Kelly have developed a set of indicators for country-level vulnerability to climate change under nine categories, including health, governance and technology. These categories are expressed in terms of multiple variables and operationalized by using proxies, such as maternal mortality per 100,000 individuals, internal refugees (1000s) scale by population and R&D investment (as a percentage of Gross National Product) [43]. Their study has shown that decadal mortality as an outcome of climate-related disasters is particularly correlated with several health vulnerability indicators (e.g., maternal mortality, calorific intake and life expectancy at birth), as well as government effectiveness and accountability. These findings point out that Sub-Saharan Africa is the most vulnerable region in the world against climate-related mortality. Furthermore, McMichael has underlined numerous health risks posed by population displacements and conflicts due to land degradation and freshwater shortages [44].

Abdussalam et al. have projected that future temperature increases in the Sahel region due to climate change can surge meningitis cases annually by 47 percent even in modest scenarios [45]. Moreover, Olago et al. have demonstrated that the surge of cholera epidemic in Lake Victoria Basin is closely linked to climate variability. They noted that the lack of clean water, sanitation, food and early warning systems make respective populations vulnerable against cholera in these regions. Olago et al. has concluded that effective adaptation strategies must involve significant improvements in health infrastructure and governance [46]. Steady has added that women are commonly excluded from decision-making processes in regard to climate change, although they are usually the first to detect the adverse effects of climate on food production, child mortality and health [47].

Employing principal component analysis and a stage-structured model, Pascual et al. have found that the impact of climate change on the expansion of malaria disease in East African Highlands was significant for the period 1950–2002 [48]. While malaria and dengue are spread to humans by mosquitos, the former disease can also affect animals. Martens et al. have projected that climate change will not only worsen public health in the currently infected areas but also expand malarial parasite into new regions. They have underlined that the level of socioeconomic development, population growth and control measures are vital factors in this process [49]. Tompkins and Di Giuseppe have demonstrated that monthly/seasonal temperature and rainfall predictions could be used as early warning systems against malaria at least 1 month in advance for Africa [50].

Chang et al. have indicated that dengue fever, which is another deadly mosquito-borne disease especially for children, will potentially affect 4.1 billion people in the world by 2055 due to humidity changes, population growth and migration. Rainfall variability due to climate change will be affecting vulnerable populations in any case: increased rainfall and flooding will create new breeding grounds for mosquitos, whereas they will find safe havens at water storages in dry regions. Since there are still no proven vaccine against dengue fever, significant improvements in sanitation and health-care infrastructures are needed to increase the resilience of populations in Africa, Asia and Latin America [51]. Considering the limits of conventional toxicological approaches, Patz and Balbus have proposed a global climate change risk assessment by incorporating geographic information system (GIS) [52].

### 2.4. Violent Conflict

Violent conflicts have various root causes, including population pressures, economic conditions and military capabilities [53,54]. Violence and forced migration may trigger one another, since civilians fleeing war zones may clash with local communities in their transit and destination countries [55,56]. For example, Percival and Homer-Dixon have provided a strong case for how scarce environmental resources interacted with broader political, socioeconomic and physical constraints and produced civil conflict in South Africa in the early years of the post-apartheid era [57]. Similarly, Mazo has called the Darfur conflict (Sudan) as the first modern climate-change conflict, which escalated due to drought and scarce natural resources, among other reasons [58]. Introducing climate risk into the picture, Hendrix and Glaser have established a causal relationship between increased rainfall and decreased probability of civil conflict in Africa, but they did not find a strong link between climate change and violent conflict in general [59].

Gleick has contributed to this debate by demonstrating that climate change and scarce freshwater resources made an adverse effect on socioeconomic conditions in Syria and became a major driver of the outbreak of civil war [17]. In parallel, Burke et al. have projected that temperature increases will bring a 54 percent surge in armed conflict occurrence by 2030 [60]. Finally, Nel and Righarts have found a strong causal relationship between natural disasters (e.g., earthquake, tsunami, flood and heat wave) and violent civil conflicts for the period 1950–2000 [61]. Yet, Buhaug has found a weak relationship between these variables in Africa, where poverty appears as a stronger predictor of conflict [62].

Folami and Folami have made an important contribution to this debate by documenting that climate change has the potential of aggravating ethnic tensions, which can undermine economic development in turn. In fact, Easterly and Levine have found that ethnically diverse countries tend to face low schooling, underdeveloped financial systems, poor infrastructure and dysfunctional foreign exchange markets, all of which hinder growth. They have concluded that ethnic group rivalry perpetuates rent-seeking behavior, since each group tries to divert scarce resources on themselves through public policies [63]. Based on experiments and surveys in Uganda, Habyarimana et al. have also found that the absence of shared ethnicity and social sanctions lead individuals to defect (in their contracts, etc.) more than otherwise [64]. As for foreign aid, Nielsen et al. have shown that a sudden and sharp decline in aid revenues significantly increases the risk of violent civil conflict, since recipient governments have less resources to distribute and sustain military forces [65].

### 2.5. Governance and Institutions

The quality of governance is critical for the adaptive capacity of societies facing climate change and other human security challenges. Acemoglu, Johnson and Robinson have provided empirical evidence for the impact of mortality rates of colonizers on the type of colonial institutions: although high mortality rates led to extractive institutions, lower mortality rates opened the way to settlement of sizable civilian populations in the Americas [66]. As a proponent of the institutionalist view over the structural (geography) explanation, Acemoglu has suggested that the lack of good institutions, which involve property rights, checks and balances, rule of law and opportunities of education and economic activity for broad segments of the society, are the primary cause of income disparity among countries in the world. He supported this view by pointing out that more developed areas/countries in the 15th century (e.g., Central America where the Aztecs and the Maya ruled) are much poorer now than the (once backward) North America. Acemoglu has concluded that this “reversal of fortune” could not be explained without taking different institutions created by European colonialists into account [67] (p. 28).

Furthermore, Raleigh, Jordan and Salehyan have looked into a number of cases in which labor migration followed sudden or chronic disasters. They have indicated that government policies, such as establishing safer relied camps and encouraging rural investment through remittances, have a substantial impact on the form of population displacements (e.g., seasonal or permanent) and the relief conditions of potential refugees [68].

When institutional factors are added into the picture, foreign aid becomes even more controversial. The negative effect of foreign aid on the likelihood of democratization during the Cold War has been demonstrated by several studies [69]. Furthermore, Kono and Montinola have found in their study of 123 countries that continued foreign aid help autocratic leaders survive, as they use part of these external resources to prevail over crises. Moreover, aid conditionality is ineffective in promoting economic reforms (on average) for autocratic leaders. Based on these findings, the authors have recommended that foreign aid not be given to countries led by autocrats unless there is an emergency or a disaster situation [70].

Baccini and Urpelainen have investigated the relationship between donor behavior and preferential trade agreements (PTAs), which typically offer benefits from trade while prescribing economic reforms in developing partners. They have found that, since economic reforms typically hurt several segments of a society in the short-run, the US and the EU tend to provide more foreign aid to developing countries during their adjustment with the PTAs. However, this PTA-aid relationship only exists for developing countries that are in the process of democratization, which increases their credibility to abide by their contractual agreements [71].

Overall, poverty, disease, violent conflict and political regime type are highlighted as major drivers of human migration in the literature. In response to these challenges, policymakers have employed foreign aid with less than stellar performances. In the following section, we operationalize our variables and develop our hypotheses in regard to the impact of various aspects of human security on LSFM in Africa.

## 3. Materials and Methods

In this study ordered logistic regression analysis is performed on 48 African countries for the period 2011–2017 (lagged 1-year). Ordered logistic regression model is a category of logistic regression model in which the dependent variable category is not a continuous but an ordered set (i.e., the relative ordering of response values is known but the exact distance between them is not known). In the literature, this type of a model is sometimes referred to as the logit model or the cumulative link model. The ordered logistic model is only applicable to datasets where the dependent variable has a distinct order with at least two or more categories. Examples of suitable datasets for this analysis include socioeconomic status (low, medium and high) or LSFM, as it is used on a three point scale in this study. The definition of this scale is given below.

Logistic regression and ordered logistic regression utilize different methodologies in the calculation of the probabilities of ordered dependent data sets. In that sense, one has to pay significant attention to the definition of orders that will be used in the analysis of an ordered data set. For example, logistic regression application assigns probabilities that a variable will take on a specific value of an ordered set. As such it is more suitable for applications where the orders in the dataset are well defined and categorically distinct. However, ordered logit approach assigns probabilities to values that will fall below a certain threshold of an ordered set. Given this difference, the logit model is more suitable for datasets where the differences among orders are less distinct. It is our observation that the dataset we are working with belongs to this second category of data sets and thus logit model is preferred in this study. However, the logit model and the results that are obtained from using it can sometimes be difficult to understand for laypersons. For example the coefficient of determination or root mean square criteria do not exactly correspond to the standard scales that are used in linear regression analysis to determine if there is a good fit [72].

The assumptions for the use of a logit model are as follows [72]: (i) the dependent variable is measured on an ordinal level; (ii) one or more of the independent variables are either continuous, categorical or ordinal; (iii) multicollinearity should not exist among the independent variables. Logistic regression requires that there to be little or no multicollinearity among the independent variables. This means that the independent variables should not be too highly correlated with each other. If there is a mild correlation, means of its effect need to be evaluated on the outcome. Finally, (iv) proportional odds—i.e., each independent variable has an identical effect at each cumulative split of the ordinal dependent variable. These assumptions should be tested, and if there is a violation to these assumptions and this violation is not correctable, the logit model approach would not be the proper choice of analysis. We have tested our dataset against these four criteria and have seen that only the multicollinearity criteria may be violated if the independent and control variables are not chosen properly. In our analysis below, we have paid specific attention to this problem and chosen our variables accordingly.

In this study, the unit of analysis is the “country-year”, and our spatial parameter includes all continental African countries (i.e., island states and the disputed Western Sahara were excluded). The dependent variable is large-scale forced migration, which we have coded on a 3-point scale as “0—Below 1%”, “1—Between 1% and 5%, inclusive” and “2—Greater than 5%”, where percentages are calculated over the total population of a country. The dependent variable includes the total number of refugees, asylum-seekers and all other types of internally and externally displaced persons originated in a country, as provided by the UNHCR. Since we investigate forced migration, we did not include voluntary migrants in our dataset. Furthermore, we use a proportional indicator (percent of total population) for LSFM rather than thousands of people, since 1 million forced migrants would be a relatively small number compared to Nigeria’s population of 181 million, whereas it would make up almost one fifth of Eritrea, thus indicating different levels of human insecurity.

One of our basic assumptions is that large-scale forced migration (LSFM) is a product of severe human insecurity in countries of origin, whether the reason is food shortage (e.g., the Irish famine of the 1860s), violent conflict (e.g., Syrian civil war) or something else. As we discussed earlier, this is a primary reason for why we have pooled all forced migrants, whether their legal status is refugee, asylum-seeker or internally displaced person. Destination countries may categorize these persons differently, but the concern of this study is that they are all forced migrants. Accordingly, we have employed the UNHCR Population Statistics Reference Database in order to compute the total number of asylum-seekers, refugees (including refugee-like situations, as described by the UNHCR), and IDPs for each African country of origin, regardless of their destination countries, for each year (2012–2017, inclusive).

The independent variable is climate risk. As discussed in the literature review, the African continent is exposed to a variety of climatic zones. In order to capture this diversity in climatic effects and the outcomes it may trigger, we include all continental African countries from several sub-regions and analyze the situation in each region as a function of its climatic effects. Considering the lack of consistent time-series climate data at sub-country level, we use Busby et al.’s classification of physical exposure to climate-related hazards. Using historic data, they mapped physical exposure as an outcome of climatic conditions (e.g., droughts, floods and cyclone winds), as well as population density and other factors, as of 2011 (final entry in their study) [23]. The gist of this approach is that the same degree of physical exposure experienced in densely populated areas is rated as higher risk than that in sparsely populated areas (e.g., an uninhabited desert with increasing temperatures does not signify high climate risk for humans). Based on Busby et al.’s study, we put African countries into three categories: High Climate Risk (i.e., high physical exposure and high/mid-level population density—3), Mid-Level Climate Risk (i.e., low physical exposure and high/mid-level population density—2), and Low Climate Risk (i.e., low physical exposure and low population density—1) (see Appendix A). The reason for choosing the period 2011–2017 as our temporal parameter is to ensure maximum data availability, since UNCHR’s migration data (currently) ended in 2017.

Furthermore, this study employs five control variables: violence, political regime, life expectancy at birth (as a proxy for public health), income per capita and official development assistance. Violence is an aggregated category in which higher scores signify higher intensity (in terms of fatalities) for all interstate, civil and ethnic conflicts involving a country, as provided by the Major Episodes of Political Violence, 1946–2017 dataset [73]. The political regime data are provided by the Polity IV Annual Time-Series 1800–2017 dataset, where the Autocracy-Democracy scale ranges from −10 (strongly autocratic) to +10 (strongly democratic) [74]. Extracted from the World Bank database, income per capita (PPP, international USD) and life expectancy at birth (in years) are other control variables that are directly related to human security. Finally, official development assistance (ODA) per capita (current USD) is tested as a potential remedy for LSFM [30]. Our hypotheses are developed as the following:

**H1**. *The higher the climate risk in a country, the higher the likelihood of large-scale forced migration in this country*.

**H2**. *The higher the aggregated level of civil and interstate violence involving a country, the higher the likelihood of LSFM in this country*.

**H3**. *The lower the level of democracy in a country, the higher the likelihood of LSFM in this country. (A limitation of this hypothesis is that, in extremely autocratic cases like former East Germany (GDR) or present-day North Korea, large-scale emigration may practically be impossible due to the heavily guarded borders)*.

**H4**. *The shorter the life expectancy at birth in a country, the higher the likelihood of LSFM in this country*.

**H5**. *The lower the income per capita in a country, the higher the likelihood of LSFM in this country*.

**H6**. *The higher the official development assistance per capita received by a country, the lower the likelihood of LSFM in this country*.

We present our statistical findings in the following section.

## 4. Results

The ordered logistic regression analysis (using odds ratios for interpretation) in Model 1 indicates that civil/interstate violence and political regime type are the most powerful predictors of large-scale forced migration in Africa (see Table 1). Their directions support Hypothesis 2 and Hypothesis 3: the higher the aggregated level of civil and interstate violence (H2) and the lower the level of democracy (H3), the higher the likelihood of LSFM in a country. Given the literature, these results are not surprising, since violence and repression are considered as primary causes of forced migration.

Poverty provides support for Hypothesis 5, although its effect is small. People living in poor or extremely poor conditions might have strong motivations to migrate, but their impoverishment might serve as a hindrance against their mobility at the mass scale. Climate risk and life expectancy at birth are not statistically significant in Model 1. Before elaborating on these results, we introduce the findings from Model 2.

The violence variable was omitted in Model 2 while keeping the other variables from the first model intact. The reason is that, if adverse climatic conditions create and/or escalate violent conflicts as proposed in the literature, running both variables simultaneously on LSFM could make climate risk statistically insignificant. In fact, Model 2 indicates that climate risk becomes a strong predictor of LSFM once the violence variable is removed (although the model suffers from explanatory power as a whole, as pseudo R2 signifies). Life expectancy at birth, which is affected not only by public health but also by violent conflicts, becomes significant in Model 2 as well. In both cases, the directions of the relationship provide support for Hypothesis 1 and Hypothesis 4: the higher the climate risk (H1) and the lower the life expectancy at birth (H4), the higher the likelihood of LSFM in a country.

Finally, the full model (Model 3) introduces official development assistance (ODA) as a potential remedy for LSFM. In this case, violence and political regime type still preserve their dominance, whereas the thinly supported income loses its significance. In all three models, a one-point increase in the Autocracy-Democracy scale (towards democratization) reduces the odds of LSFM by about 10 percent. The ODA result is particularly interesting, because higher ODA actually increases the likelihood of LSFM, although marginally. This result can give weight to the longstanding criticism that several donor countries have provided generous aid packages to authoritarian and corrupt regimes for their own national security objectives without paying much attention to human security consequences in recipient countries [75].

Overall, our findings indicate that intense civil and interstate conflicts trigger LSFM in African countries. This factor, especially its interstate dimension, falls under the national security domain. On the other hand, democracy, which is considered vital in providing political, personal and community security, is a human security-related factor that affects mass population displacements. Public health and poverty are also significant in some models, thus indicating further impact of human security on LSFM. Foreign aid should be handled with caution, since uncoordinated and misused aid can do more harm than good, as demonstrated in the literature.

Finally, climate risk has an indirect effect on LSFM, but it is hard to differentiate its cumulative effect from violent conflicts within short time periods. Yet, it is important to note that Mozambique, which is listed in our High Climate Risk category (see Appendix A) and did not experience LSFM during the period 2011–2017, has been hit by two cyclones within only two months in 2019 and already has 1 million affected children [76]. Thus, climate change is possibly (and unfortunately) in the process of becoming more significant in people’s lives and in human security statistics.

## 5. Discussion: Responding to Human Security Challenges in Africa

Our findings indicate that climate change indirectly affects large-scale forced migration by igniting violent conflicts, although not all conflicts are climate-related for sure. These results run parallel to a recently published article by Abel et al., who have found that climatic conditions increased the likelihood of conflict for Western Asian countries (e.g., Syria) and led to a surge in asylum seeking for the period 2010–2012 [77]. Thus, policies that address LSFM need to take climate change mitigation and adaptation into account. Otherwise, conventional measures, such as aerial bombing of terrorist groups or imposing restrictive measures against undemocratic governments (e.g., EU sanctions against Somalia, South Sudan and Sudan) alone are unlikely to stop large-scale forced migration in Africa [78].

In its first Climate Change Action Plan (CCAP; 2011–2015), the African Development Bank (AfDB) has declared, “Climate change is depleting Africa’s renewable natural land, water, and forest resources. The impact of climate change is further aggravated by fast population growth in Africa, resulting in an increasing demand on natural resources” [79] (p. iii). In its second CCAP (2016–2020), the AfDB has renewed its stark warnings and added, “One issue that continues to be alarmingly persistent however, is the significant disparity between financing for adaptation and financing for mitigation” [80] (p. 1). Noting the limited funding as a primary concern, the Food and Agriculture Organization (FAO) of the UN has also emphasized the necessity of securing food resources for the livelihoods of host communities and displaced people in Lake Chad Basin, which is threatened by the joint effect of natural hazards, adverse climate conditions and violent conflicts [81]. The World Bank and the UN Environment Programme have estimated that around USD 7.5 billion would be needed annually to finance Africa’s adaptation to 2 °C warming, but the current financing adds up to a only 10% of this amount [82]. Considering these climate risks, Collier, Conway and Venables have provided a number of adaptation strategies, such as switching to climate-resilient cultivation practices [22]. Surely, there is a delicate balance between mitigation, adaptation and agricultural productivity. For instance, rice cultivation may lead to clearing new lands and deforestation, whereas livestock production is a primary source of greenhouse gas emissions. Yet, novel tools like climate index insurance are already being used by small farmers in Senegal [83].

Although foreign aid is a controversial tool, it cannot be totally disregarded due to the urgent needs of developing countries. Tol has put forward that development aid could reduce vulnerability of African communities in certain areas (e.g., sanitation and food production) while increasing it in other areas (due to rising energy consumption and air pollution). Accordingly, Tol has suggested that funding for climate adaptation could be more effective if it is channeled into mitigation, especially into emission reduction, in developed countries, which are primarily responsible for Africa’s climate change problems [84]. Finally, national and non-governmental solutions (e.g., microfinance) can become part of the solution, as demonstrated by the Nobel Peace Prize winner Prof. Muhammad Yunus and Grameen Bank in Bangladesh [85]. Overall, policy planning for human security needs to follow an interdisciplinary approach in order to comprehend the full scale of the challenges and to develop effective responses.

## 6. Conclusions

Large-scale forced migration (LSFM) poses a significant humanitarian, political and socioeconomic challenge for the origin, transit and destination countries. Addressing the causal link between human security and LSFM, we employ the most recent data and present in this study a quantitative analysis of 48 African countries for the period 2011–2017. Our findings indicate that civil and interstate conflicts, lack of democratic institutions and poverty are among the primary causes of LSFM, whereas climate change has an indirect effect on the dependent variable. On the other hand, foreign aid is a poor instrument in alleviating the migration pressure at its source.

Our findings indicate that the conventional understanding of security is not sufficient to address the LSFM in the 21st century. Instead, policymakers need to take human security into account in order to complement national security with governance, public health, environmental and other aspects. In fact, the causal link between climate risks and LSFM is likely to become more significant in the upcoming decades.

A major limitation of interdisciplinary studies of this kind is that natural and human-related factors may involve different time-lags. For instance, a violent conflict may lead to LSFM within a year, whereas the adverse effects of climate change may accumulate over several years. Thus, the study of their joint effects within the same model remains a challenge. As for policy recommendations, we suggest that policymakers place human security at the core of their threat analyses in order to develop effective responses to the massive and unprecedented migrant flows in Africa and other parts of the world.

## Figures and Tables

**Table 1:** Ordered logistic regression (odds ratios presented) of large-scale forced migration.

Variable	Model 1	Model 2(w/o Violence)	Model 3(w ODA)
Climate risk	1.276	5.922 **	1.271
	(0.397)	(0.338)	(0.402)
Violence	2.459 **		2.524 **
	(0.131)		(0.133)
Political Regime	0.868 **	0.901 **	0.862 **
	(0.041)	(0.036)	(0.040)
Life expectancy	0.970	0.907 **	0.969
	(0.035)	(0.030)	(0.034)
Income	0.999 *	0.999 *	0.999
	(0.000)	(0.000)	(0.000)
Official development asst.			1.008 *
			(0.004)
*N*	272	272	272
Pseudo R2	0.329	0.151	0.336

Note: Standard errors in parenthesis, * *p* < 0.1, ** *p* < 0.05, (Prob > chi2 = 0.00).

## Appendix A

**Table A1:** Climate Risk (countries categorized according to their historical physical exposure to climate-related hazards and population density in affected areas) [23].

High Risk (2)	Mid-Level Risk (1)	Mid-Level Risk (1) (cont’d)	Low Risk (0)
Algeria	Benin	Liberia	Botswana
Angola	Burkina Faso	Malawi	Gabon
Central African Rep.	Burundi	Mali	Namibia
DR Congo	Cameroon	Mauritania	Rep. of Congo
Egypt	Chad	Niger	
Eritrea	Cote d’Ivoire	Nigeria	
Ethiopia	Djibouti	Rwanda	
Libya	Equatorial Guiena	Senegal	
Morocco	Eswatini (Swaziland)	Sierra Leone	
Mozambique	Gambia	South Africa	
Somalia	Ghana	Togo	
South Sudan	Guiena	Uganda	
Sudan	Guiena-Bissau	Zambia	
Tanzania	Kenya	Zimbabwe	
Tunisia	Lesotho		
